# Artificial Intelligence and Emotional Support: A Comparative Study of University Students with and Without Disabilities

**DOI:** 10.3390/healthcare14010075

**Published:** 2025-12-27

**Authors:** Raquel Suriá-Martínez, Fernando García-Castillo, Carmen López-Sánchez, José A. García del Castillo

**Affiliations:** 1 Faculty of Economics and Business, University of Alicante, 03690 Alicante, Spain; 2 Department of General Didactics and Specific Didactics, University of Alicante, 03690 Alicante, Spain; fgarciadelcastillo@ua.es; 3 Department of Communication and Social Psychology, University of Alicante, 03690 Alicante, Spain; mc.lopez@ua.es; 4 Department of Health Psychology, University of Miguel Hernandez, 03202 Elche, Spain; jagr@umh.es

**Keywords:** artificial intelligence, students with disabilities, university, academic support, emotional support, informational support, inclusion, educational technology

## Abstract

**Background/Objectives**: This study explores the perceived usefulness and use of artificial intelligence (AI)-based technologies as informational and emotional support among university students with and without disabilities. **Methods:** The sample consisted of 358 students from the University of Alicante, aged between 16 and 30 years; 88 participants identified as having a disability, while 270 reported no disability. The gender distribution was balanced, with 53% women and 47% men. An ad hoc questionnaire was applied to evaluate familiarity, frequency of use, and perceived usefulness of AI as a means of obtaining informational and emotional support. **Results:** The results of the multivariate analyses indicated that students without disabilities reported greater familiarity with and more frequent use of AI tools compared to students with disabilities. Significant differences were found in perceived usefulness for obtaining both informational and emotional support, with higher ratings among students without disabilities, and a moderate effect size. Moreover, frequency of use was positively associated with perceived usefulness in both groups, suggesting that practical experience influences users’ evaluations. **Discussions:** The findings suggest that students perceive AI as a useful resource for informational and emotional support. However, as with other technologies, addressing accessibility and perception gaps is crucial to promote effective inclusion in university settings.

## 1. Introduction

Currently, artificial intelligence (AI) has become one of the pillars of digital transformation, rapidly and quietly integrating into multiple human activities. Its presence influences how people process information, make decisions, manage academic tasks, and participate in social environments, acting as a mediating agent with a direct impact on educational contexts [1,2,3]. Recent advances have enhanced adaptive, conversational, and empathetic capabilities, expanding AI’s potential to intervene in traditionally sensitive areas, such as emotional support or personalized learning experiences [4,5,6].

Consequently, AI has become an emerging resource in academic settings, both for resolving doubts and for study organization, emotional support, and autonomous knowledge acquisition. In this regard, AI is increasingly recognized as a tool for informational and emotional support among students [2,3]. Conversational systems, virtual assistants, and generative model-based platforms allow students to obtain quick and contextualized responses, resolve academic questions, and access personalized explanations that complement formal learning. Additionally, these tools offer spaces for emotional expression, stress regulation, and perceived support, especially during times of uncertainty or academic overload. While they do not replace human intervention, they represent a hybrid resource combining information, guidance, and emotional containment, whose usefulness may vary depending on accessibility, trust in technology, and student characteristics [4,5].

Accumulated empirical evidence supports this trend. In educational settings, Rajesh. [3] demonstrated that intelligent tutoring systems (ITSs) improve both academic performance and self-regulated learning through immediate and adaptive feedback. Franganillo [2] highlighted that AI enables the personalization of educational trajectories through continuous analysis of student progress. Other studies, such as Kose et al. [7], have shown that AI-based assistants can enhance student motivation and reduce cognitive load in complex tasks. Similarly, Liu and Liu [5] observed that AI dialog tutors foster self-regulated learning and deeper content comprehension among university students.

In terms of emotional support, Fitzpatrick et al. [8] demonstrated that the chatbot “Woebot” reduced mild anxiety and depression symptoms among young people after two weeks of use, while Inkster et al. [9] found that “Wysa” improves emotional well-being and stress management by providing coping strategies based on cognitive behavioral therapy principles. More recently, Fulmer et al. [10] showed that AI-based conversational assistants can enhance perceived social support, particularly among students experiencing high levels of academic stress.

However, evidence indicates that AI use is neither homogeneous nor universally beneficial. Perceived usefulness, trust in these systems, and integration into daily life depend on multiple personal, social, and cultural factors [11,12]. These differences are particularly significant among students with disabilities, who face additional challenges compared to their peers without disabilities. Barriers are not only physical or technological but also include cognitive, sensory, and emotional accessibility obstacles that hinder effective interaction with AI systems. For example, unintuitive interfaces, content not adapted to specific needs, or conversational models lacking sensitivity to particular limitations can restrict autonomy and cause frustration. When these needs are not considered in the design and implementation of tools, there is a risk of digital exclusion and exacerbation of pre-existing inequalities, affecting academic participation, personal autonomy, and access to informational and emotional support resources [13,14,15,16].

For students with disabilities, AI can represent both an opportunity and a challenge. On one hand, familiarity with digital technologies can promote empowerment, inclusion, and well-being [17,18]. On the other hand, accessibility limitations and variability in adaptive capacities of conversational models can restrict autonomous use, affect perceived usefulness, and increase vulnerability to isolation [19,20,21]. In contrast, students without disabilities generally have better access, skills, and participation in digital environments, facilitating more natural and frequent integration of AI tools into their academic and personal lives [22,23]. Comparing both groups helps identify technological, perceptual, and experiential gaps, which are essential for advancing inclusive design from early development stages [24,25].

From a scientific perspective, this analysis requires a solid theoretical framework to interpret these differences. Models such as the Technology Acceptance Model (TAM), the Unified Theory of Acceptance and Use of Technology (UTAUT), and Human-Centered AI approaches provide guidance for understanding how variables like perceived usefulness, ease of use, algorithmic trust, and accessibility influence technology adoption, especially among vulnerable populations. Integrating these approaches allows for building a coherent theoretical model that ensures conceptual clarity and methodological rigor.

In this context, the present study proposes a comparative analysis between students with and without disabilities to examine how they perceive AI as an informational and emotional support tool and to what extent frequency of use relates to this perception. It also explores whether this relationship is bidirectional—i.e., whether continued use promotes more positive perceptions or whether initial perceptions drive more frequent use—in order to provide empirical evidence that contributes to more inclusive and equitable AI technology design.

Based on these considerations, this work aims to contribute to a deeper and more nuanced understanding of the role AI can play in the emotional and communicative lives of young people, particularly those historically marginalized in the design of emerging technologies.

The specific study objectives are

1. To design and validate a questionnaire developed to measure the perceived usefulness of artificial intelligence (AI) in providing informational and emotional support to university students with and without disabilities.
2. To describe the level of familiarity and current use of AI technologies among higher students with and without disabilities.
3. To compare perceived usefulness between university students with disabilities and those without disabilities.
4. To analyze the relationship between perceived usefulness and frequency of AI use in both groups of university students.

Based on previous literature and the study objectives, the following hypotheses were proposed:

5. The questionnaire assessing the perceived usefulness of AI for informational and emotional support demonstrates adequate psychometric properties and is valid for measuring these dimensions in university students with and without disabilities.
6. University students without disabilities report higher levels of familiarity with and use of AI-based tools than students with disabilities.
7. University students without disabilities perceive greater usefulness of AI compared to students with disabilities.
8. There is a positive relationship between perceived usefulness and frequency of AI use in both groups of university students.

## 2. Materials and Methods

### 2.1. Participants

The study was conducted with a sample of 358 university students aged 18 to 30 (M = 22.4; SD = 4.1). Of the total sample, 88 participants (24.6%) identified as having a disability, while 270 (75.4%) reported no disability. Among students with disabilities, the distribution by type was as follows: motor disability, 34.1% (n = 30); psychosocial disability, 20.5% (n = 18); hearing impairment, 15.9% (n = 14); intellectual disability, 18.2% (n = 16); and visual impairment, 11.3% (n = 10).

The sample was selected through collaboration agreements with two universities in Alicante, Spain. Recruitment followed inclusion criteria requiring participants to be enrolled students, of legal age, and, in the case of those with disabilities, to have an officially recognized condition. Students without disabilities were required not to have requested academic accommodations. Individuals who were not enrolled at the time of data collection, were unable to complete the study instruments, or chose not to participate voluntarily were excluded. A balance in key sociodemographic variables, such as sex and age, was ensured to facilitate comparisons between the two groups.

Regarding sex, 53.1% of the sample were women (n = 190) and 46.9% were men (n = 168), with this proportion maintained in both the group with disabilities and the group without disabilities. The average age was 22.1 years (SD = 4.2) in the group with disabilities and 22.5 years (SD = 4.1) in the group without disabilities.

Participants were enrolled in various academic disciplines. The most represented fields were Social Sciences (30.2%), Health Sciences (24.3%), Engineering and Technology (22.6%), Economics and Business (13.7%), and Arts and Humanities (9.2%). The distribution across fields was similar in both groups, reinforcing the comparative homogeneity of the sample (Table 1).

### 2.2. Instruments

To assess young people’s interaction with artificial intelligence (AI), an ad hoc questionnaire specifically designed for the present study was used. The instrument consisted of a 20-item questionnaire, structured into three main dimensions: AI Familiarity (5 items), Frequency of AI Use (5 items), and Perceived Usefulness of AI (10 items), the latter subdivided into informational support and emotional support.

All items were rated using a 5-point Likert scale, with higher values indicating greater familiarity, usage frequency, or perceived usefulness (Table 2).

### 2.3. Procedure

Data collection was carried out over a three-month period (September to November) in 2025, with the approval of an institutional ethics committee and the informed consent of all participants. Participation was voluntary, and participants were previously informed about the objectives of the study, data confidentiality, and the possibility of withdrawing at any time without consequences.

Recruitment was conducted by disseminating the study through institutional emails, internal university communication platforms, social media announcements, and direct invitations in classrooms or extracurricular activities. This strategy ensured the inclusion of students with diverse academic profiles and types of disabilities.

The questionnaires were administered primarily online through a secure link hosted on the university’s virtual campus, which facilitated flexible and autonomous access for most students. For participants with disabilities who required additional support, particularly individuals with visual, intellectual, or psychosocial disabilities, the questionnaires were adapted into accessible formats and completed at the Student Support Center, where they filled them out under supervision with personalized assistance when necessary. Each session, both in-person and online, was supervised to address questions and ensure response quality.

The questionnaire took approximately 20 to 30 min per participant to complete, depending on the need for additional assistance. The collected data were stored securely and confidentially, in compliance with standards for personal information protection and research ethics.

### 2.4. Data Analysis

To process and analyze the data collected through the questionnaire, SPSS statistical software (version 23.0) was used, applying various statistical techniques to meet the study’s objectives.

In the first place, a descriptive analysis of sociodemographic variables and the main questionnaire indicators was conducted, including frequencies, percentages, means, and standard deviations, in order to characterize the sample and to examine the level of familiarity, frequency of use, and perceived usefulness of AI as an informational and emotional support resource among young people with and without disabilities. Although the questionnaire items were based on a 1-to-5 Likert scale, these variables were treated as continuous in the statistical analyses. This decision is grounded in the fact that Likert scales with five or more response categories are widely considered robust approximations of interval-level measurement, enabling the assumption that the distances between response options are sufficiently equidistant to justify the use of parametric techniques. The methodological literature has demonstrated that, under these conditions, treating such data as continuous yields reliable and valid estimates, particularly when calculating means, variances, and linear relationships among variables.

To compare differences between university students with and without disabilities, multivariate analyses of variance (MANOVA) were conducted. This technique was used, first, to jointly examine the variables of perceived familiarity and frequency of use, and second, to assess differences in perceived usefulness, considering both its informational and emotional dimensions, between the two groups of students.

The use of MANOVA was justified by the presence of multiple conceptually related dependent variables, which allows the effect of group membership to be evaluated in an integrated manner while accounting for potential correlations among the variables and reducing the risk of Type I error associated with conducting multiple independent univariate tests. Moreover, this technique is appropriate when comparing groups across several psychological or perceptual dimensions simultaneously.

When significant effects were identified, effect size was examined using partial eta squared (η^2^p) in order to estimate the practical magnitude of the differences observed between groups. According to conventional criteria, values close to 0.01 were interpreted as small effects, around 0.06 as medium effects, and values near 0.14 as large effects.

Additionally, to explore the relationship between perceived usefulness and frequency of use of AI-based technologies, Pearson correlation analysis was applied, considering the strength and direction of the association. Linear regression analyses were also conducted to assess whether frequency of use could predict perceived usefulness in each group, as well as to identify possible moderating or mediating variables.

These techniques make it possible not only to determine statistical differences between young people with and without disabilities, but also to examine the functional relationship between frequency of use and perceived usefulness, providing relevant evidence for the design of inclusive interventions and technologies

## 3. Results

- Objective 1. To design and validate a questionnaire developed to measure the perceived usefulness of AI in providing informational and emotional support to students with and without disabilities.

To assess the perceived usefulness of artificial intelligence (AI) in informational and emotional support, a specific questionnaire was developed. Initially, a pool of items was generated based on a review of the existing literature on the use of AI in educational and student support contexts. Each item was subsequently evaluated through an expert judgment process to assess its clarity, relevance, and adequacy to the construct, applying inclusion and exclusion criteria to ensure the appropriateness of the questions. Following this process, 20 items were selected, which constituted the final instrument used to assess students with and without disabilities.

To empirically evaluate the adequacy of the instrument, an exploratory factor analysis (EFA) was conducted using a subsample of 85 students. The suitability of the correlation matrix was examined using the Kaiser–Meyer–Olkin (KMO) measure (KMO = 0.91) and Bartlett’s test of sphericity (χ^2^(190) = 4980.3, *p* < 0.001), confirming that the data were appropriate for factor analysis. Subsequently, an EFA was performed using the maximum likelihood extraction method with Oblimin rotation, which identified four factors: AI familiarity, frequency of AI use, informational usefulness, and emotional usefulness. The total variance explained by the model was 86%, distributed as follows: familiarity (27%), frequency of use (24%), informational usefulness (17%), and emotional usefulness (18%), (Table 3).

As shown in Table 4, the items cluster coherently according to the expected factors, and factor loadings above 0.60 indicate that the items contribute substantially to their respective dimensions.

The positive and moderate correlations among factors indicate that, although related, each factor captures a distinct construct.

Subsequently, a confirmatory factor analysis (CFA) was conducted to test a four-correlated-factor model. The model fit indices were as follows: χ^2^/df = 1.98, CFI = 0.971, TLI = 0.965, RMSEA = 0.039, and SRMR = 0.035. These values indicate an excellent model fit and confirm the validity of the theoretical structure, particularly supporting the distinction between the two subdimensions of perceived usefulness (informational vs. emotional).

To ensure item stability and internal homogeneity, Cronbach’s alpha and composite reliability (CR) were calculated (Table 5).

All factors exceeded the recommended threshold of 0.70, indicating high internal consistency and reliable measurement of each construct. Overall, these results support the adequacy of the questionnaire for assessing AI familiarity, frequency of use, and perceived usefulness in both academic and emotional support contexts among university students with and without disabilities.

- Objective 2: To describe the level of familiarity and current use of AI technologies among students with and without disabilities.

To address this objective, the independent variable was disability status (yes = 1, no = 0), and the dependent variables were (a) perceived familiarity with AI (measured on a scale from 1 to 5) and (b) frequency of AI use in daily life (ordinal scale transformed into a continuous variable).

The results revealed statistically significant differences between the two groups in both variables. The multivariate effect was significant—Wilks’ Λ = 0.94, F(2, 355) = 11.40, *p* < 0.001, and partial η^2^ = 0.06—indicating that the set of dependent variables differed between groups.

Subsequent univariate analyses showed that students with disabilities reported significantly lower levels of perceived familiarity (M = 3.3, SD = 1.0) compared to students without disabilities: (M = 3.8, SD = 0.9), F(356) = 10.74, *p* < 0.05, and partial η^2^ = 0.06.

Similarly, regarding frequency of use, the group of students with disabilities obtained lower scores (M = 3.0, SD = 0.9) than their peers without disabilities (M = 3.6, SD = 0.8): F(356) = 6.92, *p* < 0.05, and partial η^2^ = 0.03 (Table 6).

- Objective 3: Compare perceived usefulness (informational, emotional, and overall academic support) between students with disabilities and those without disabilities.

The multivariate effect was significant—Wilks’ Λ = 0.95, F(3, 354) = 6.27, *p* < 0.001, and partial η^2^ = 0.05—indicating that the overall pattern of dependent variables differed between students with and without disabilities.

Subsequent univariate analyses revealed significant differences across all evaluated dimensions. For informational support, students with disabilities reported lower scores (M = 3.4, SD = 0.9) than students without disabilities: (M = 3.7, SD = 0.8), F(356) = 4.78, *p* < 0.05, and partial η^2^ = 0.04.

Regarding emotional support, the group of students with disabilities also showed lower scores (M = 2.9, SD = 1.0) compared to their peers without disabilities: (M = 3.2, SD = 0.9), F(356) = 3.86, *p* < 0.05, and partial η^2^ = 0.03.

Finally, for overall academic usefulness, students with disabilities reported lower values (M = 3.15, SD = 0.85) than students without disabilities: (M = 3.48, SD = 0.78), F(356) = 4.98, *p* < 0.05, and partial η^2^ = 0.04 (Table 7).

- Objective 4: Analyze the relationship between perceived usefulness and frequency of AI use in both groups.

To examine this objective, Pearson correlations were calculated to examine the relationship between frequency of AI use and perceived usefulness. In the total sample (N = 358), a significant positive correlation was found: r = 0.42, *p* < 0.001. Similarly, students without disabilities (n = 270) showed a stronger significant positive correlation (r = 0.45, *p* < 0.001), whereas among students with disabilities (n = 88), the relationship was also positive but more moderate (r = 0.29, *p* = 0.007). Overall, these results suggest that higher frequency of AI use is associated with higher perceived usefulness, although the strength of this relationship varies by group.

Subsequently, a moderated linear regression analysis was conducted to examine whether disability moderates the relationship between frequency of use and perceived usefulness. The model included frequency of use, group (0 = without disability, 1 = with disability), and the Frequency × Disability interaction as predictors.

Regression assumptions were assessed through graphical analyses of residuals. The plot of residuals versus fitted values showed random dispersion around zero, with no evident patterns, indicating homoscedasticity. Residuals ranged approximately from −2.5 to 2.8 (M = 0.01, SD = 0.91). The Q–Q plot of residuals showed close alignment with the reference line, suggesting normality, with approximate skewness of 0.03 and kurtosis of 2.9. Additionally, the histogram of residuals displayed a symmetric distribution centered around zero, further confirming that the assumptions of normality and homoscedasticity were adequately met.

The full model explained 27% of the variance in perceived usefulness (R^2^ = 0.27) and was statistically significant, F(3, 354) = 43.56, *p* < 0.001. Frequency of use positively predicted perceived usefulness (β = 0.38, t = 7.72, *p* < 0.001). Disability also had a negative effect on perceived usefulness (β = −0.15, t = 2.88, *p* = 0.004), indicating that students with disabilities perceived AI as less useful compared to students without disabilities. Finally, the interaction between frequency of use and disability was significant (β = −0.19, t = 2.33, *p* = 0.021), suggesting that the relationship between frequency of use and perceived usefulness is stronger among students without disabilities, thereby evidencing a moderating effect of disability status (Table 8).

## 4. Discussion

The relevance of this study lies in its contribution to understanding how university students, both with and without disabilities, interact with artificial intelligence (AI) in their daily lives. In a context where AI has become an omnipresent and necessary tool, it is essential to analyze not only the level of access and use, but also the perception that these groups have regarding its usefulness. This work creates a space for reflecting on the opportunities and challenges that AI presents in terms of inclusion and the principles of universal accessibility [13,14].

The results show that university students, both with and without disabilities, use AI as an informational and emotional resource, but significant differences exist in familiarity, frequency of use, and perceived usefulness.

Regarding the first objective, which was to develop and validate a questionnaire aimed at assessing the perceived usefulness of artificial intelligence (AI) in providing informational and emotional support to students with and without disabilities, a thorough review of the existing literature [3,4,5,6,11,14,26,27,28] was conducted prior to item creation. This review ensured that the questions were both relevant and representative of the constructs to be measured. Additionally, the content validity of the instrument was supported through the evaluation by four experts in education, educational technology, and inclusion, combined with clear inclusion and exclusion criteria for items, resulting in a final set of 20 items deemed appropriate and representative.

The exploratory factor analysis (EFA) confirmed the proposed dimensional structure, identifying four coherent factors: AI familiarity, frequency of AI use, informational usefulness, and emotional usefulness, which together explained 86% of the total variance. The suitability of the data for factor analysis was supported by an excellent KMO index (0.91) and a significant Bartlett’s test of sphericity, indicating that the correlation matrix was appropriate for this type of analysis. The variance explained by each factor and the clear grouping of items suggest that the questionnaire is consistent and capable of capturing meaningful differences in AI perceived usefulness among students.

These findings are in line with previous studies on instrument development in educational contexts, which emphasize the importance of combining expert judgment and factor analysis to ensure the validity and reliability of questionnaires [3,25]. The identification of specific dimensions allows for a more detailed evaluation of AI’s impact on informational and emotional aspects, providing guidance for future pedagogical interventions and technology inclusion strategies. Overall, the results indicate that the questionnaire is a valid and reliable instrument, suitable for subsequent studies examining the perceived usefulness of AI and its relationship with other educational variables.

Regarding the second objective, understanding perceived familiarity and use of AI, the results indicate that students without disabilities reported significantly higher levels of familiarity with and frequency of use of AI-based tools. This is consistent with previous studies showing that people with disabilities face greater barriers to access and usability in digital environments [15,16,25]. Although inclusive digital solutions have been developed, structural and contextual obstacles persist, limiting equitable adoption, particularly affecting students with sensory, psychosocial, or intellectual disabilities [17].

This finding reinforces the need to analyze familiarity and use separately, since familiarity reflects knowledge and confidence in the technology, whereas use captures actual practice and integration into academic and personal activities [22,23]. Students with disabilities, although familiar with AI, show lower frequency of use, suggesting that cognitive exposure does not always translate into real interaction, likely due to usability or design barriers.

Regarding the third objective, the perceived usefulness of AI, the results show that students without disabilities provided higher ratings in both informational and emotional dimensions, as well as in overall academic usefulness. These results can be explained by their higher frequency of use and familiarity, which reinforces the perception of academic benefits, learning support, and emotional accompaniment. In this context, studies such as those by Fitzpatrick et al. [8], Inkster et al. [9], and Fulmer et al. [10] show that perceived effectiveness of AI systems is linked to user experience and interaction accessibility.

Meanwhile, students with disabilities showed moderate perceptions: they recognize the potential value of AI, although their experience is more limited. The results obtained are consistent with the recent literature showing that students with disabilities often maintain moderate views about AI, acknowledging its potential but also its limitations. For example, Melo-López et al. [24] report that AI can significantly enhance accessibility through personalization, format conversion, and automated supports, although they warn of gaps in infrastructure and teacher training that shape user evaluations. Similarly, Li et al. [25] conclude that students with diverse educational needs perceive clear benefits in autonomy and adaptive learning, but also express concerns about system reliability and the lack of institutional guidance. Likewise, Panjwani-Charania and Zhai [29] show that although tools such as intelligent tutors or reading-support systems facilitate learning for students with specific difficulties, the evidence remains limited and heterogeneous, generating a cautious stance toward adoption.

Finally, regarding the fourth objective, the study explored the relationship between perceived usefulness and frequency of AI use. Correlation and moderated regression analyses showed a positive relation between frequency of use and perceived usefulness in both groups. However, when examining this relationship using moderated regression, the effect was significantly weaker in the group with disabilities. This can be interpreted as a warning sign: in this group, frequent use does not always translate into a better experience or positive perception, possibly due to accessibility barriers or unmet expectations when turning to AI [17,18]. This finding aligns with previous studies indicating that virtual assistants or AI systems require adaptations and personalization for users with specific needs to obtain real benefits [8,9].

Although AI can serve as a valuable informational and emotional resource, intensive use may also have less beneficial effects [7,27,28]. Frequent use of AI systems for emotional support can generate dependency, reducing students’ autonomy in regulating emotions and seeking human support. Ho et al. [27] note that AI-based emotional companions, although helpful in providing comfort, may induce excessive use or affective dependency, limiting the development of real social and emotional skills.

Similarly, using AI as an informational resource may foster cognitive overload or technological dependency, where students rely more on automated recommendations than on their own critical judgment [28,30,31]. Among students with disabilities, these risks may be greater if AI becomes the primary resource due to accessibility barriers in traditional academic environments. Therefore, AI should be implemented in a supervised and complementary way, promoting autonomy, critical thinking, and balanced use to maximize benefits and minimize risks of dependency or social isolation [28,30,32,33].

Taken together, these results demonstrate that while AI holds significant potential as an informational and emotional resource, its equitable use depends on inclusive and accessible digital environments. Incorporating diverse perspectives from the earliest stages of technological design not only enhances the experience of those facing obstacles, but also contributes to building a fairer, more accessible, and sustainable academic environment for the entire university community. Furthermore, the empirical evidence supports the need for educational and technological policies that consider functional, cognitive, and psychosocial diversity, ensuring that frequent use of AI translates into real and equitable benefits—aligned with the idea that perceived inequalities are social and environmental, not inherent to users’ disabilities.

Nonetheless, although this research offers valuable information, it is important to acknowledge certain limitations that may have influenced the findings. First, as a cross-sectional study, we were only able to observe associations at a specific moment; therefore, it is not possible to establish causal relationships or understand how perceptions may change over time. Future research should include longitudinal designs to analyze the evolution of perceptions and AI use as technologies increasingly integrate into educational settings. Participation was also voluntary, meaning that those who chose to respond may have had particular interest or experience with AI. This may have introduced bias, as we do not know whether non-participants would hold different views or usage patterns—thus limiting generalizability. Future studies could employ more representative sampling strategies or mixed methods, such as in-depth interviews or intentional sampling, to capture voices less visible or less familiar with these technologies.

It is also important to consider that the group of young people with disabilities was smaller than the group without disabilities. This limits the ability to make strong generalizations and, additionally, the study did not differentiate among types of disabilities. Therefore, we cannot determine whether, for example, students with visual disabilities have different experiences from those with intellectual or hearing disabilities. Future research should increase the sample size of students with disabilities and analyze the particularities of each disability type, incorporating intersectional approaches to identify specific needs and develop truly inclusive AI tools. Finally, the study was conducted in a very specific context: university students in a particular region and cultural setting. This limits the possibility of extrapolating the findings to young people at other educational levels, of differing ages, or in other cultural environments. Thus, multicenter or cross-cultural studies are recommended to understand how sociocultural and contextual factors influence the perception and adoption of AI.

These limitations do not diminish the value of the findings, but they do invite cautious interpretation and further exploration through broader and more diverse research designs.

From a practical perspective, the results of this study help identify key areas for intervention. First, the strategic role of educational institutions is highlighted in democratizing access to AI tools—not only by providing technology but also through training activities that promote critical digital literacy and awareness of ethical and inclusive use. Second, it is essential that AI developers, particularly those designing applications for informational, educational, or emotional support, integrate cognitive, sensory, and affective accessibility criteria from the earliest design stages. This includes not only adapting interfaces but also reviewing language, interaction formats, response times, and availability of alternative communication channels.

Likewise, the potential of AI as an emotional support resource deserves deeper consideration. Although these technologies do not replace professional mental health intervention, they can play an important complementary role, especially as bridges to specialized services or as everyday support tools. For this to be beneficial for young people with disabilities, interactions with these systems must not be perceived as another source of stress or exclusion. In this sense, truly empathetic AI must necessarily be accessible AI.

Lastly, the findings open up multiple avenues for future research. It is essential to deepen the analysis of differences by type of disability, as the experience of a young person with a visual disability is not comparable to that of someone with a psychosocial or cognitive condition. It would also be valuable to include variables such as technological self-efficacy, trust in AI, or level of social support, all of which may modulate the relationship between use, perception, and satisfaction. To continue advancing the development of these tools in inclusive contexts, future research should adopt interdisciplinary approaches, consider the diversity of user profiles, and explore psychological, technological, and social variables that influence perception and use of AI. Only then will it be possible to ensure that the opportunities offered by artificial intelligence do not paradoxically become new forms of exclusion.

## 5. Conclusions

This study confirms that AI is perceived as a useful resource by university students with and without disabilities, both in its informative dimension and in its role as emotional support. However, the differences observed in perceived usefulness and frequency of use show that significant barriers still remain that limit equitable use of these technologies.

The positive relationship identified in this study between perceived usefulness and frequency of use reinforces the importance of improving the interaction experience in order to increase effective adoption. In this regard, future strategies should focus on removing technical and design obstacles, fostering inclusion from the development stage, and promoting a technological culture that is sensitive to functional diversity.

To continue advancing knowledge and application of these tools in inclusive contexts, it is suggested that new lines of research be promoted that integrate interdisciplinary approaches, consider diverse profiles, and delve into the psychological, technological, and social variables that influence the perception and use of AI. In this way, it would be possible to ensure that the opportunities offered by artificial intelligence do not paradoxically become new forms of exclusion.

## Figures and Tables

**Table 1:** Sociodemographic characteristics of the sample by disability status.

	Variable	Total (n = 358)	With Disability (n = 88)	Without Disability (n = 270)
Gender	Women	190 (53.1%)	47 (53.4%)	143 (53.0%)
	Men	168 (46.9%)	41 (46.6%)	127 (47.0%)
Age (M, SD)		22.4 (4.1)	22.1 (4.2)	22.5 (4.1)
Field of study	Social Sciences	108 (30.2%)	27 (30.7%)	81 (30.0%)
	Health Sciences	87 (24.3%)	20 (22.7%)	67 (24.8%)
	Engineering and Technology	81 (22.6%)	20 (22.7%)	61 (22.6%)
	Economics and Business	49 (13.7%)	12 (13.6%)	37 (13.7%)
	Arts and Humanities	33 (9.2%)	9 (10.2%)	24 (8.9%)

**Table 2:** Dimensions and items of the questionnaire on familiarity, frequency of use, and perceived usefulness of artificial intelligence (AI).

Dimension	Items
1. Familiarity with AI	- How familiar do you feel with the term artificial intelligence? Are you familiar with using applications that use AI? Have you used applications or platforms with AI? - Do you consider that you understand how AI-based technologies work? - Have you received information or training about AI? - How often do you seek information about AI advancements?
2. Frequency of AI use	- Use of virtual assistants (Siri, Alexa, Google Assistant) - Use of chatbots for inquiries or digital support - Use of AI-based wellness or mental health apps - Use of AI for educational or study-related tasks - Use of AI for entertainment or socialization
3. Perceived usefulness of AI	Informational support - AI helps me obtain useful information quickly - AI-generated responses are clear and accurate - I use AI to complement my learning or studies - AI facilitates access to hard-to-find resources - AI improves my academic or personal productivity Emotional support - AI provides me with emotional support when I need it - I feel comfortable expressing emotions through AI - AI responses to emotional topics are empathetic and appropriate - AI can help reduce feelings of loneliness or isolation - I prefer using AI for certain emotional topics rather than talking to people

**Table 3:** Factor loadings (EFA—4 factors, Oblimin rotation).

Item	F1 Familiarity	F2 Frequency	F3a Informational Usefulness	F3b Emotional Usefulness
General familiarity	0.78	0.12	0.05	0.04
AI use	0.74	0.21	0.09	0.07
AI understanding	0.81	0.10	0.18	0.09
AI training	0.69	0.05	0.11	0.08
AI information search	0.63	0.28	0.14	0.12
Virtual assistants	0.18	0.72	0.20	0.15
Chatbots	0.09	0.77	0.13	0.11
Well-being apps	0.16	0.69	0.31	0.22
AI for studying	0.12	0.81	0.25	0.18
AI for entertainment	0.03	0.68	0.10	0.12
Fast information	0.04	0.11	0.74	0.09
Clear answers	0.10	0.12	0.77	0.08
Complements studying	0.05	0.29	0.79	0.12
Access to resources	0.08	0.18	0.75	0.09
Improves productivity	0.12	0.15	0.71	0.11
Emotional support	0.05	0.21	0.10	0.67
Express emotions	0.14	0.10	0.11	0.72
Empathetic responses	0.11	0.09	0.12	0.69
Reduces loneliness	0.03	0.28	0.14	0.65
Preference for emotional AI	0.15	0.12	0.13	0.62

**Table 4:** Correlations among factors (EFA).

Factor	F1 Familiarity	F2 Frequency	F3a Informational Usefulness	F3b Emotional Usefulness
F1 Familiarity	1	0.52	0.55	0.48
F2 Frequency	0.52	1	0.60	0.54
F3a Informational usefulness	0.55	0.60	1	0.62
F3b Emotional usefulness	0.48	0.54	0.62	1

**Table 5:** Internal consistency and composite reliability.

Factor	Number of Items	Cronbach’s α	Composite Reliability (CR)
F1 Familiarity	5	0.86	0.87
F2 Frequency	5	0.84	0.85
F3a Informational usefulness	5	0.88	0.89
F3b Emotional usefulness	5	0.85	0.86

**Table 6:** Comparison of perceived familiarity and frequency of AI use by disability status.

Dependent Variable	Group	M	SD	95% CI	F	*p*	Partial η^2^
Perceived Familiarity	With disability	3.3	1.0	3.1–3.5	10.74	0.001	0.06
	Without disability	3.8	0.9	3.7–3.9			
Frequency of Use	With disability	3.0	0.9	2.8–3.2	6.92	0.009	0.03
	Without disability	3.6	0.8	3.3–3.8			

**Table 7:** Comparison of AI perceived usefulness by disability status.

Dimension	Group	M	SD	95% CI	F (1, 356)	*p*	Partial η^2^
Informational support	With disability	3.4	0.9	3.2–3.6	4.78	0.030	0.04
	Without disability	3.7	0.8	3.6–3.8			
Emotional support	With disability	2.9	1.0	2.7–3.1	3.86	0.050	0.03
	Without disability	3.2	0.9	3.1–3.3			
Overall academic usefulness	With disability	3.15	0.85	3.0–3.3	4.98	0.027	0.04
	Without disability	3.48	0.78	3.4–3.6			

**Table 8:** Correlations between AI frequency of use and perceived usefulness, and moderated regression analysis.

Analysis/Predictor	r/β	t	*p*
Pearson correlations			
Total (N = 358)	0.42	—	<0.001
Without disability (n = 270)	0.45	—	<0.001
With disability (n = 88)	0.29	—	0.007
Moderated linear regression			
Frequency of use	0.38	7.72	<0.001
Disability (0 = no, 1 = yes)	−0.15	2.88	0.004
Frequency × Disability	−0.19	2.33	0.021
Model	R^2^ = 0.27	F(3, 354) = 43.56	<0.001

Note. The correlations show positive relationships between perceived usefulness and frequency of use, which are stronger among students without disabilities. The moderated linear regression indicates that disability moderates the relationship between frequency of use and perceived usefulness. The model explains 27% of the variance in perceived usefulness and is statistically significant.

## Data Availability

The data are not available because the database used contains sensitive information related to students, including data associated with disability conditions.
